# Stamping out cancer

**DOI:** 10.1038/sj.bjc.6601486

**Published:** 2003-12-09

**Authors:** U Sanyal

**Affiliations:** 1 Department of Anticancer Drug Development, Chittaranjan National Cancer Institute, Calcutta 700026, India

**Keywords:** stamps, cancer awareness, prevention, fund raising

## Abstract

It is universally acknowledged that if cancer is to be controlled, prevention and down staging are essential. In the year 2000 about 10 million new cases were registered, while 6.3 million people died from cancer worldwide. Stamps are regarded as a very useful and educative tool in fighting cancer by creating awareness and raising money for treatment and research. This year (2003) is the seventy-fifth anniversary of the issue of the first anticancer stamps in 1928, so an up-to-date review of the field of oncophilately is timely.

Cancer is posing a serious social and economic problem globally since it was estimated by various organisations including IARC (2001) that in the year 2000 about 10 million new cases were registered while about 6.3 million people died from cancer worldwide. It appears that by 2020, cancer will kill over 10 million people globally. An Austrian stamp issued in 1976 describes the threat of death due to cancer (stamp 1).

It is necessary to take effective preventive measures and cancer awareness among the general population is essential. It is universally recognised that the anticancer stamps can play an excellent role in various ways as to create awareness and to collect money for treatment and research. This is because philately or stamp collection is still one of the most popular hobbies. Due to their universal circulation and appeal, stamps are powerful and effective messengers. It is also possible to use stamps issued for some other purposes in describing various aspects of cancer as will be exemplified later on. Only one report (Taub, 1978) and two cover illustrations (Cover Illustration, 1977; Sanyal, 2002) have so far been published in journals, to our knowledge, on oncophilately, a term used by us to describe the branch of philately dealing with oncology. Hence it is thought relevant to present up-to-date knowledge of anticancer and related stamps in the 75th year after the first stamp issue.

## HISTORY OF CANCER STAMPS

Five Swedish stamps on King Gustaf V issued in 1928 are regarded as the first anticancer stamps because the entire amount collected from sales was donated for cancer control (stamp 2). This was followed by a set of three Danish stamps issued in 1929 (stamp 3). Norway is the third country with a semipostal issue in 1931 (stamp 4) in the aid of Norwegian Radium Hospital. Since then, numerous countries have published several regular or semipostal stamps (stamps 5–8). Record shows that by the year 1964, 46 countries had issued 110 stamps (Taub, 1978) and by the year 2000 about 100 countries have issued over 250 stamps. So far, Panama has issued the maximum number of stamps (approximately 30) mostly between 1939 and 1949 with the pictures of the Marie and Pierre Curie. Starting from 1992, Macedonia has issued a number of stamps. The crab being the universal symbol of cancer, stamps have been printed with pictures of decorated and ordinary crabs attacked by various weapons and instruments (stamps 9–12).

## COMMON CANCERS

Stamps are available on lung cancer (stamp 13), which is the most common cancer in terms of both incidence (1.25 million new cases; 12.5% of world total) and mortality (1.19 million deaths – 17.8% of the world total combining both males and females) in 2000 (IARC, 2001). It is the commonest cancer among males and is directly linked with smoking (85–90% of all cases occur among smokers) (stamps 14 and 15).

**Figure 1:**
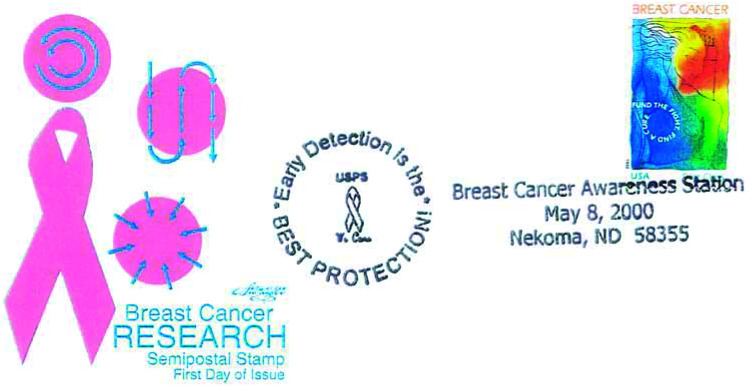
First day cover of US semipostal breast cancer stamp.

Breast cancer is the most prevalent cancer in the world among females (1 million new cases; 10.5% of world total) in 2000 (IARC, 2001). Many stamps are available on breast cancer some of which are exhibited (stamps 16–19). The US Postal Service (USPS) department has created history in the field of fund raising by issuing over 300 million semipostal breast cancer awareness stamps since 29 July 1998 (Figure 1

). Over $ 23 million dollars had been collected by March 2002 from the sale of this stamp (http://www.usps.com/news, 2002).

**Figure 2:**
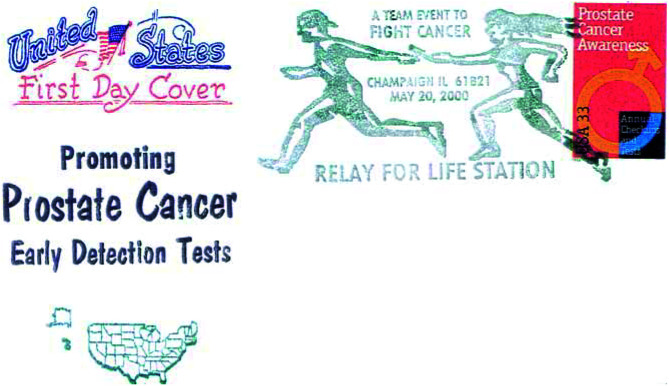
First day cover of US prostate cancer stamp.

Stamps have also been published on other cancers like prostate cancer (Figure 2

), leukaemia (stamp 20), etc.

## CAUSES OF CANCER

Doll and Peto (1981) described the relative quantification of the environmental contributions of a variety of factors such as diet, tobacco, alcohol, occupation, radiation, etc. towards its development.

### Habits

#### Tobacco

Among the carcinogenic hazards, tobacco use in the form of smoking or smokeless as chewing, etc. has been identified to be the most important one, since about 33% of all cancers are tobacco-related. Smoking has been firmly linked not only to lung cancer but also to oral, oesophageal, bladder, pancreas, cervical, nasal, stomach cancers, etc. attributed to the presence of about 40 carcinogens among 4000 other chemicals found in cigarette smoke. To date, about 65 countries have issued over 100 antismoking stamps with a view to tobacco control (stamps 21–23). Passive smoking is also very dangerous. Evidence suggests that nonsmoking women married to smokers experience an excess risk of developing lung cancer in the order of 20%. A Japanese stamp may be used to illustrate this aspect (stamp 24).

#### Alcohol

Excessive drinking causes liver, oral, pharynx, larynx and oesophageal cancers, and may increase the risks of colorectal and breast cancers. Simultaneous drinking and smoking habits are much more dangerous. Alcoholic beverages consumption potentiates the effects of tobacco smoking on cancers of the mouth, pharynx, oesophagus and larynx and has been estimated to account for about 3% of all cancer deaths. Stamps issued by many countries warn people about the use of alcohol (stamps 25 and 26).

### Exposure to carcinogenic chemicals and occupational cancer

The British Surgeon Dr Percivall Pott demonstrated the earliest example of occupational cancer in 1775, showing a greater incidence of scrotal cancer among young chimneysweepers who were exposed to tar. Lord Shaftesbury, the great social reformer, worked for the well-being of these poor lads. A British stamp is available in his memory, depicting the hand of a chimneysweeper holding the brush (stamp 27). The stamp showing a picture of aniline (stamp 28) illustrates the need for care to be taken to minimise exposure to this chemical, because as early as in 1895, Rehn was the first to observe increased rate of bladder cancers among aniline dye workers. The stamp 29 describing the structure of benzene may be similarly used to show the need to take care in handling benzene, since it is proved to be a potent human carcinogen causing leukaemia with a latency period of about 15–20 years. It is widely used as a solvent in leather, petroleum and other industries. Polyaromatic hydrocarbons (PAH) are found in exhaust gases from automobiles run by petrol and diesel, from railway locomotives and in smoke, dust and exhaust gases from various factories. It is advisable to use protection to prevent inhaling of exhaust gases from automobiles as described in a Swedish stamp (stamp 30).

### Exposure to radiations

#### Ionising radiation

The relation between ionising radiation from various sources including radioactive substances such as _92_^235^U (stamp 31) and development of cancer dates from early observations on survivors of the atomic bombs in Japan.

#### Ultraviolet radiation

**Figure 3:**
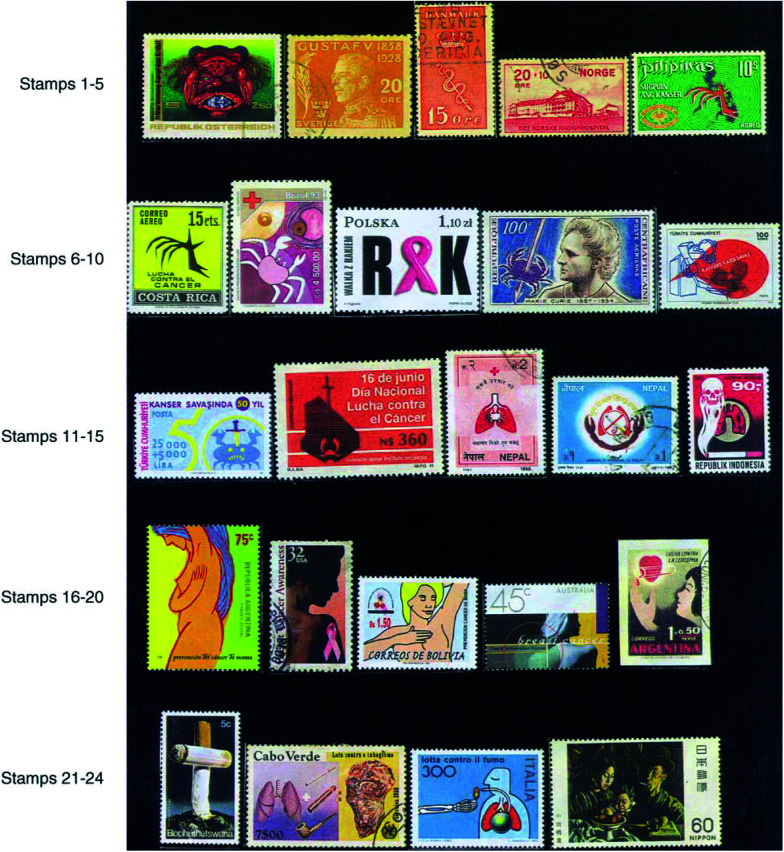

Ultraviolet radiation from sunlight is the major risk factor for skin cancers and that of lip. Stamps showing sunrays and flares (stamp 32) may be used to make people aware of the dangers of overexposure to sunrays.Figure 3

### Some viral infections

Links have already been established between some viral infections and the development of cancers such as Epstein–Barr virus and Burkitt lymphoma, hepatitis B and C and hepatocellular (liver) carcinoma, human papilloma virus (HPV) and cervical cancer in females, human immunodeficiency virus (HIV). AIDS patients infected with HIV have higher incidence and death rates from malignancies like Kaposi's sarcoma, non-Hodgkin's lymphoma, etc. Hence awareness regarding HIV infection is very important from a cancer preventive perspective also. So far about 65 countries have issued over 120 anti-AIDS stamps (e.g. stamps 33–36).

## TREATMENT OF CANCER

**Figure 4:**
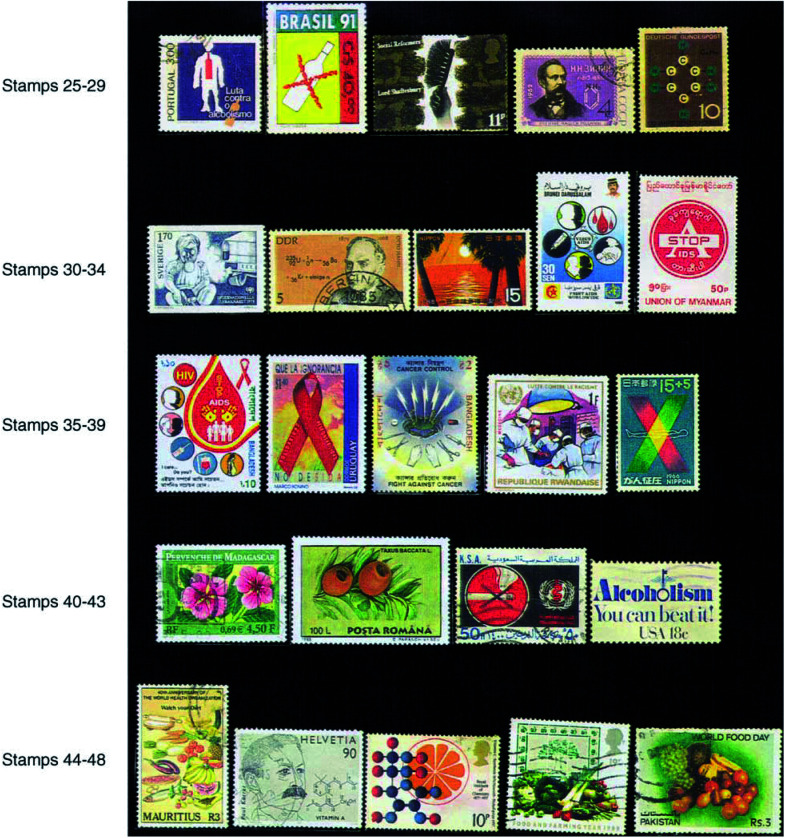

Nowadays, three chief treatment modalities – surgery, radiotherapy and chemotherapy – are used in all sorts of combinations. Bangladesh has issued a beautiful stamp depicting the picture of a crab attacked with these three modes of treatment (stamp 37). One can find individual stamps on surgery (stamp 38), radiotherapy (stamp 39) and chemotherapy. Anticancer drugs are obtained by chemical synthesis or from natural sources such as plants. A French stamp on the Madagascar periwinkle plant (*Catharanthus rosea*) may be used to point out that some important alkaloidal drugs like vincristine and vinblastine have been isolated from the plant (stamp 40). Taxol has been isolated from *Taxus brevifolia* (Pacific Yew tree) and *Taxus baccata* (Himalayan yew tree) (stamp 41).Figure 4

## PREVENTION OF CANCER

A number of reports (Byers *et al*, 2002) have been published on cancer prevention that can further be pictorially described by various stamps.

### Habits not recommended

1. Stop smoking of cigarettes, cigars, etc. (stamp 42) and using ‘smokeless’ tobacco products like crude leaves, snuff, etc.
2. Limit alcohol consumption to a minimum, if at all (stamps 26 and 43).

### Foods recommended

Diet has a vital role in the prevention of about 30–40% of all cancers (Byers *et al*, 2002). Hence it is important to watch one's diet as recommended in a Mauritius stamp issued on the occasion of WHO World Health day (stamp 44).

1. Both vitamin A (stamp 45) and vitamin C (stamp 46) are considered as antioxidants and possess potential cancer chemopreventive properties by inhibiting precancerous changes and damages of DNA leading to cancer. *β*-Carotene present in various yellow, green or orange fruits and vegetables is converted to vitamin A in the liver. Hence it is recommended to eat fresh vegetables and fruits rich in these substances and vitamins like carrot, beans, gourd, broccoli, cauliflower, tomato, etc. (stamp 47) and a variety of fruits (stamp 48).
2. The protective effects of dietary fibre present in vegetables against a number of cancers like colorectal, oesophageal and stomach have been demonstrated. Hence it is advisable to eat vegetables rich in roughage like jackfruit (stamp 49), beans (stamp 47), ladies finger, etc.
3. The medicinal plant *Curcuma longa* (stamp 50) is the source of turmeric, which contains the yellow natural colouring agent namely curcumin which may have chemopreventive properties (Krishnaswamy and Polasa, 2001).
4. Constituents of *Allium sativum* commonly known as garlic (stamp 51) have been shown in lab studies to have antitumour as well as tumour promotion inhibitory activities. Onions and ginger have also been shown to possess similar properties.
5. Tea is a beverage made from the leaves of the plant species *Camellia sinensis* (stamp 52). Tea is one of the most ancient and next to water the most widely consumed liquid in the world. Many phytochemicals including polyphenols catechin compounds like epigallocatechin gallate (EGCG), etc. present in tea leaves have shown potential chemopreventive and antioxidant properties.

Figure 5

**Figure 5:**
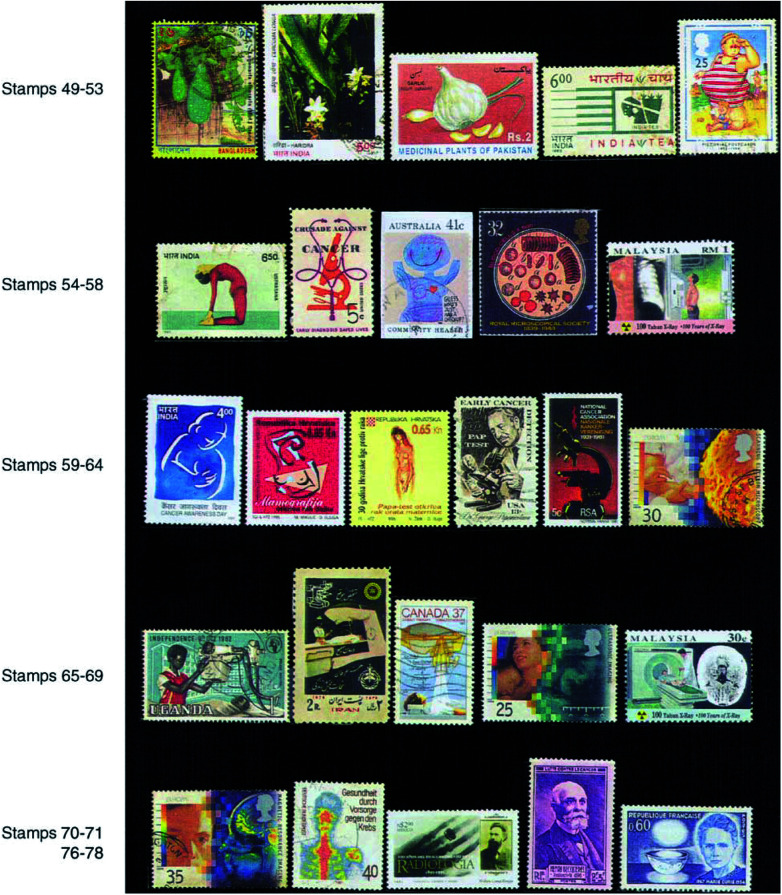

Figure 6

**Figure 6:**
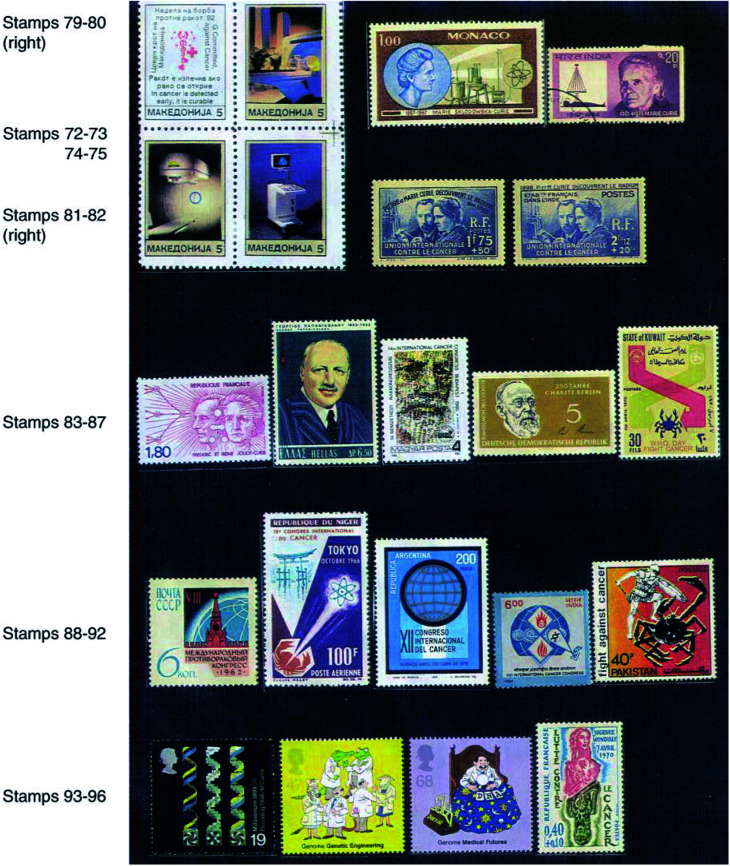

### Lifestyle

#### Body weight

Being very underweight or overweight increases the risk of cancer. Obesity (stamp 53) is associated with the endometrium, breast, kidney, colorectal and gallbladder cancers. Thus it is advisable to maintain a healthy body weight.

#### Physical activity

An active lifestyle is recommended. Physical activity decreases the risk of colon and breast cancers. Walking, cycling, gardening, household chores, climbing the stairs, practising yoga (stamp 54), etc. are recommended.

## DETECTION OF CANCER

Early detection of cancer is very important to save lives (stamp 55). Annual check-up (stamp 56) of oral cavity, skin, lymph nodes, routine examinations of blood for detection of any abnormalities in its constituents (stamp 57) and chest X-ray (stamp 58) are internationally accepted tests (ACS Monograph, 2000) for early detection in asymptomatic people. Occult blood examination in stool and urine may be recommended for older people.

*For women*: Breast awareness is important (Indian stamp 59) and clinical breast examination by a physician may be helpful where there is no national screening programme. It is also suggested to carry out mammography (stamp 60). Cervical PAP test (stamps 61 and 62) should be carried out in adult females at regular intervals.

*For men*: Population-based prostate-specific antigen (PSA) testing can reduce mortality in countries where urological follow-up and treatment are available. US Postal Service issued a semifiscal stamp in 1998 on this aspect (Figure 2).

## INSTRUMENTS AND DIAGNOSTIC TOOLS

Postage stamps describing various instruments may be used to demonstrate their application in the diagnosis, research and treatment of cancer as microscope (stamps 55 and 63), electron microscope (stamp 64), X-ray instruments (stamp 65), radiotherapy instruments (stamps 66 and 67), ultrasonography (USG) (stamp 68), computed topographic (CT) scanning (stamp 69), magnetic resonance imaging (MRI) (stamp 70), nuclear medicine imaging (scintigraphy) (stamp 71), mammography, etc. In 1992, Macedonia had brought out a set of four stamps with the message of early cancer detection in one of them and with the picture of diagnostic equipment in the other three (stamps 72–75).

## EMINENT PERSONALITIES

Many scientists and physicians appear on stamps for their outstanding contribution in the fight against cancer. WC Roentgen (1845–1923) – discoverer of X-rays in 1895 and the first recipient of the Nobel prize in 1901 (stamp 76). Henri Becquerel (1852–1908) (stamp 77) – discoverer of the phenomenon of spontaneous emanations later termed radioactivity by Madame Marie Curie. Radiotherapy has originated from this discovery. Madame Marie Curie (1867–1934) and Pierre Curie (1859–1906) – world-renowned French scientists who discovered radium in 1898, which was the first radioactive substance used for the treatment. Madame Curie appeared in about 100 stamps either alone (e.g. stamps 9, 78–80) or with her husband Pierre Curie (stamps 81 and 82). In 1938, France and its 21 colonies issued the latter stamps (81 and 82) as a tribute to the 40th anniversary of this discovery. The surtax of these stamps was given to the International Union Against Cancer. Pierre Curie was also remembered alone in some stamps. Irene Joliot Curie (1897–1956), elder daughter of Madame Curie, and Frederic Joliot (1900–1958) son-in-law of Madame. Curie – joint discoverers of artificial radioactivity (stamp 83). Based on their discovery, a number of artificial radioisotopes have been prepared some of which find use in cancer. George N Papanikolaou (1883–1962) (stamps 62 and 84) – discoverer of PAP test for early diagnosis of cervical cancer that has saved the lives of millions of women worldwide. Moritz Kaposi (1837–1902) (stamp 85) – famous physician who identified in 1872 a special type of sarcoma that was named after him as Kaposi's sarcoma. Rudlof Virchow (1821–1902) (stamp 86) – founder of cellular pathology. He discovered and named gliomas and giant cells. He had also named many common tumours.

## WORLD-FAMOUS INTERNATIONAL ORGANISATIONS

Postage stamps and cancellations are available on world-famous organisations like International Union Against Cancer (UICC) (stamps 81 and 82), World Health Organization (WHO) (stamp 87), etc.

## INTERNATIONAL CANCER CONGRESS

Starting from 1933, UICC organises International Cancer Congress every fourth year where scientists, clinicians, nurses, etc. from all over the world meet. Special commemorative stamps have been issued on these occasions by various countries: 8th held in Russia in 1962 (stamp 88), 9th held in Japan in 1966 (stamps 39 and 89), 12th held in Argentina in 1978 (stamp 90), 14th held in Hungary in 1986 (stamp 85) and 16th held in India in 1994 (stamp 91).

## FUTURE OF CANCER RESEARCH AND TREATMENT

Thus our fight against cancer will continue till we achieve success (stamp 92). Research is ongoing globally, and newer concepts, improvements in therapeutic modalities and new drugs are being added continuously. Recent global efforts in DNA decoding (stamp 93) and human genome mapping leading to genome genetic engineering (stamps 94 and 95) will help to identify and repair the defective genes (gene therapy of cancer). The message that should reach everybody is that cancer will be defeated – sooner or later – as is beautifully demonstrated in a French stamp issued in 1970 picturing a smiling lady cured of cancer overcoming the threat of death displayed underneath (stamp 96).

## CONCLUSION

It is therefore convincingly demonstrated that, in addition to specific anticancer stamps, various other stamps can be used to describe different aspects of cancer. The significant role of stamps in raising awareness among people regarding this dreadful disease has been highlighted.
